# Cashew (*Anacardium occidentale* L.) Nuts Counteract Oxidative Stress and Inflammation in an Acute Experimental Model of Carrageenan-Induced Paw Edema

**DOI:** 10.3390/antiox9080660

**Published:** 2020-07-24

**Authors:** Marika Cordaro, Rosalba Siracusa, Roberta Fusco, Ramona D’Amico, Alessio Filippo Peritore, Enrico Gugliandolo, Tiziana Genovese, Maria Scuto, Rosalia Crupi, Giuseppina Mandalari, Salvatore Cuzzocrea, Rosanna Di Paola, Daniela Impellizzeri

**Affiliations:** 1 Department of Biomedical, Dental and Morphological and Functional Imaging, University of Messina, Via Consolare Valeria, 98125 Messina, Italy; cordarom@unime.it; 2 Department of Chemical, Biological, Pharmaceutical and Environmental Sciences, University of Messina, 98166 Messina, Italy; rsiracusa@unime.it (R.S.); rfusco@unime.it (R.F.); rdamico@unime.it (R.D.); aperitore@unime.it (A.F.P.); egugliandolo@unime.it (E.G.); tgenovese@unime.it (T.G.); gmandalari@unime.it (G.M.); dimpellizzeri@unime.it (D.I.); 3 Department of Biomedical and Biotechnological Sciences, University of Catania, Via Santa Sofia 97, 95123 Catania, Italy; mary-amir@hotmail.it; 4 Department of Veterinary Sciences, University of Messina, 98168 Messina, Italy; rcrupi@unime.it; 5 Department of Pharmacological and Physiological Science, Saint Louis University School of Medicine, Saint Louis, MO 63104, USA

**Keywords:** paw edema, cashew nuts, antioxidant, inflammation, polyphenols, analgesic

## Abstract

Background: *Anacardium occidentale* L. is a medicinal plant with powerful anti-oxidative and anti-inflammatory properties. Acute inflammatory events cause tissue alterations, decrease of anti-oxidative endogenous enzymes such as superoxide dismutase, catalase and glutathione, neutrophils infiltration, increase in the activities of myeloperoxidase, malondialdehyde, and pro-inflammatory release. Methods: Paw edema was induced by subplantar injection of carrageenan into the right hind paw in rats, but 30 min before a group of animals were orally treated with 100 mg/kg of cashew nuts to evaluate the anti-inflammatory and anti-oxidative response. Results: In the present work, we found that (1) cashew nuts reduced the development of carrageenan-induced paw edema limiting the formation of edema and pain; (2) cashew nuts ameliorated the diminutions of the anti-oxidative enzymes caused by carrageenan injection; (3) cashew nuts decreased myeloperoxidase malondialdehyde activity induced by carrageenan; and (4) cashew nuts acted by blocking pro-inflammatory cytokines response and nitrate/nitrite formation stimulated by carrageenan injection. Conclusions: The mechanisms of anti-inflammatory and analgesic effects exerted by cashew nuts were relevant to oxygen free radical scavenging, anti-lipid peroxidation, and inhibition of the formation of inflammatory cytokines.

## 1. Introduction

Inflammation is the first physiological response to tissue injury involving a complex cascade of reactions which can be provoked by numerous agents such as toxic compounds, microbes, etc., [1,2]. The changes that happen during acute inflammatory event have physiologically functions in controlling infection and restoring tissue to its normal state. The acute inflammatory state is generally composed of four sub-events distinctly into: (1) Exudation of fluid that helps deliver plasma proteins to sites of damage; (2) infiltration of neutrophils that leads to remove pathogens and cellular fragments; (3) vasodilation that the delivery of necessary proteins and cells (like exudation) and increasing tissue temperature; (4) pain and loss of function help to enforce rest and lower the risk of further tissue damage [3].

When acute inflammatory response was controlled, the result is the elimination of the infectious agents followed by a resolution and repair phase [3]. However, when the inflammation is uncontrolled it can be harmful to health [4,5,6]. Main events during the inflammatory state involve nitric oxide (NO) imbalance, lipid peroxidation, cytokines release, and maybe the most important, neutrophil-derived reactive oxygen species (ROS) formation [7].

The instability of free radicals is fundamentally the result of the loss of an electron that leads to an heightened reactivity and to a constantly “steal” electrons from other molecules starting a dangerous chain reaction called “free radical damage” [8]. Principal targets of these “steal” are proteins, lipids, and DNA/RNA, and all these modifications in different and several molecules may increase the chances of mutagenesis. In fact, ROS/RNS overproduction over a prolonged period of time can cause serious injury of the cellular structure and functions. For this reason, it is mandatory to remove it quickly [9]. Free radicals are important mediators that initiate inflammatory processes and, consequently, their neutralization by antioxidants and radical scavengers can attenuate inflammation. In order to minimize the damage caused by free radicals, the organism utilizes several enzyme such as superoxide dismutase (SOD) and catalase (CAT) and cofactor such as glutathione (GSH) [10,11]. Often, however, the physiological endogenous response put in place by the antioxidant enzymes may not be sufficient to limit ROS production [12].

Actually, the primary treatment during acute inflammatory event is the use nonsteroidal anti-inflammatory drugs (NSAIDs), in particular to limit neutrophil migration and oxygen free-radical generation, but several studies demonstrated that long-term use could lead to a lot of side effects, such as cardiovascular and gastrointestinal complications [13,14].

For this reason, it is mandatory to find new molecules to counteract novel drugs for treatment of acute inflammation and pain.

In the past decades, there has been a growing interest in studying and quantifying the antioxidant and anti-inflammatory constituents of vegetables in terms of their potential health functionality through action against inflammatory conditions [15,16]. The use of plant with known anti-inflammatory and/or antioxidant properties can be of great significance in therapeutic anti-inflammatory treatments. In particular, plants or fruit or nuts, rich in phenolic compounds are known for their wide ranges of biological activities, including anticancer, antibacterial, antioxidant, antidiabetic, and anti-inflammatory properties which could constitute an alternative in therapeutics [17].

Numerous studies, has been carried out on nuts demonstrating that a diet enriched with walnuts decreases serum cholesterol levels compared to a standard healthy diet [18]. By definition, tree nuts are dry fruits with one seed in which the ovary wall becomes hard at maturity. One of the most popular edible tree nuts is cashews (*Anacardium occidentale* L.) [18].

Cashew nuts are rich of unsaturated fatty acids (UFAs) such as oleic (ω-9) and linoleic (ω-6) acid, flavonoids, anthocyanins and tannins, fiber, folate and tocopherols [19,20,21,22,23].

The proposal of nuts as cardio-protective foods was supported from both epidemiological observations suggesting a consistent inverse association between nut intake and development of heart disease and numerous short-term clinical trials that showed beneficial effects of nut intake on the lipid profile [24,25,26,27,28]. Additionally, recent studies proved that the use of cashew nuts (*Anacardium occidentale* L.) can modulate the effects of several chronic inflammatory state such as colitis, degenerative joint disease, dyslipidemia, and others [29,30,31,32,33,34].

Its anti-inflammatory and anti-oxidative activities is probably due to the inhibition of the biosynthesis of inflammatory mediators by blocking the activities of 5-lipoxygenase (5-LOX) or cyclooxygenase 2 (Cox-2) which makes it a promising treatment for different inflammatory diseases [35,36].

However, until today, nobody evaluated its effects during acute inflammatory events.

Carrageenan-induced paw edema is a common murine experimental model used for the study of new compounds during the acute phase of inflammation [37]. As well-known, following injury induced by carrageenan, there is cell infiltration, mainly neutrophils, that contributes to the inflammatory response by producing myeloperoxidase and pro-inflammatory cytokines [4,6,30,38,39]. Moreover, a critical role during the development of the inflammatory state is the lipid peroxidation and the imbalance between ROS production and anti-oxidant enzyme activities [4,6,30,38,39]. With this background in mind, we used this consolidated experimental model to evaluate for the first time the analgesic, anti-inflammatory, and anti-oxidant effects of cashew nuts.

## 2. Materials and Methods

### 2.1. Animals

Male rats (Sprague-Dawley (200–230 g, Envigo, Milan, Italy)) were used throughout. The University of Messina Review Board for animal care (OPBA) approved the study. All animal experiments agree with the new Italian regulations (D. Lgs 2014/26), EU regulations (EU Directive 2010/63), and the ARRIVE guidelines.

### 2.2. Carrageenan-Induced Paw Edema

After anesthesia with 5.0% isoflurane in 100% O_2_ rats were subjected to a subplantar injection of CAR (0.1 mL/rat of a 1% suspension in saline) with a 27-gauge needle into the right hind paw, as described previously by Morris and Britti [40,41]. The animals were sacrificed after 6 h post CAR-injection by isoflurane overdose. All analyses were performed in a blinded manner of experimental groups [42].

### 2.3. Experimental Groups

Rats were randomly divided into the following groups:

(1) CAR + vehicle (saline): rats were subjected to CAR-induced paw edema (*n* = 10);

(2) CAR + cashew nuts (100 mg/kg): rats were subjected to CAR-induced paw edema and cashew nuts (100 mg/kg) was administered 30 min before CAR (*n* = 10);

(3) The sham-operated group underwent the same surgical procedures as the CAR group, except that saline or drugs were administered instead of CAR (*n* = 10 for all experimental groups).

The tested dose was chosen based on previous studies performed in our laboratories [30]. After sacrifice, paw tissue and blood were collected for histological and biochemical analysis.

In another sets of experiments (*n* = 6 for each group) we analyzed ROS production and 5-LOX/COX pathways.

### 2.4. Assessment of CAR-Induced Paw Edema

Edema was assessed as previously described [40]. In short, the volume of the paw was measured with a plethysmometer (Ugo Basile, Comerio, Italy) immediately before carrageenan was injected and for 6 h at hourly intervals subsequently. For each animal, edema was expressed as increase in paw volume (mL) after CAR injection relative to pre-injection value.

### 2.5. Pain-Related Behavioral Analysis in the CAR-Induced Inflammation

To evaluate the analgesic effects of cashew nuts we made a plantar and Von Frey tests. Briefly, during the plantar test we analyzed the hyperalgesic response to heat at different time point using a Basile Plantar Test (Ugo Basile, Varese, Italy) with a cut-off latency of twenty seconds to prevent tissue injury. A mobile unit containing a high-intensity projector bulb was located to carry on a thermal stimulus directly to a single hind paw from beneath the chamber. The withdrawal latency period of injected paws was determined with an electronic clock circuit and thermocouple. Results are expressed as paw withdrawal latencies [41,43]. Additionally, Von Frey test (BIO-EVF4, Bioseb, Vitrolles, France) was made. The device encloses a force transducer furnished with a plastic tip. The force applied was measured when pressure is applied to the tip. The tip was applied to the hind leg’s plantar region, and an increasing force was exerted upwards before the paw was extracted. The withdrawal threshold was defined as the force, expressed in grams, at which the rats removed the paw [41,44,45].

### 2.6. Myeloperoxidase (MPO) and Malonaldehyde (MDA) Activity

As previously described for MPO evaluation, paw tissues were homogenized in 0.5 percent hexadecyltrimethyl-ammonium bromide dissolved in 10 mM potassium phosphate buffer (pH 7.0) and centrifuged at 20,000× *g* at 4 °C for 30 min. A supernatant aliquot had been allowed to react with a 1.6 mM tetramethylbenzidine/0.1 mM H_2_O_2_ solution. The rate of absorbance shift was measured at 650 nm, using a spectrophotometer. MPO activity was defined as the amount of enzyme degrading 1 mM of peroxide at 37 °C within 1 min, and expressed in units per gram of wet tissue weight [46,47]. Additionally, for MDA analysis, paw tissues, collected at the end of experiment, were homogenized in 1.15% KCl solution. An aliquot of the homogenate was added to a reaction mixture containing sodium dodecyl sulfate (SDS), acetic acid (pH 3.5), thiobarbituric acid, and distilled water. Samples were then boiled and centrifuged. The supernatant’s absorbance was measured at 650 nm using spectrophotometry [46,47,48].

### 2.7. Determination of Nitrite/Nitrate Concentration in Paw

Levels of nitrite/nitrate production in the paw tissue were determined as previously described by Costantino et al. [47]. Briefly, at the end of experiment, paws were cut and centrifuged to recover a sample of the edematous fluid. Blood was separated from the fluid sample and nitrite + nitrate (NOx) production, an indicator of NO synthesis, was measured [47]. Concentrations of nitrate were determined by comparison of regular sodium nitrate solutions prepared in saline solution at the OD550.

### 2.8. Evaluation of Cytokines and Antioxidant Enzymes in Blood

TNF-α, IL-6, IL-1β, and IL-10 levels from each sample were measured in duplicate with highly sensitive rat Elisa kit according to manufacturer’s instructions (R&D Systems, Minneapolis, MN, USA) [49]. Additionally, also the levels of SOD, GSH, and CAT were assayed in blood according to manufacturer’s instructions (Cusabio Biotech Co., Ltd, Wuhan, Hubei, China) [50,51,52,53].

### 2.9. Histological Examination of the CAR-Inflamed Hind Paw

For histological examination hematoxylin/eosin (H/E) was made and observed blinded to the treatment protocol. Briefly, paw tissues were taken at the end of experiment, and were dehydrated, embedded in Paraplast, and cut into sections of 7 μm and observed under microscopy (Leica DM7, Milan, Italy). The gradation of inflammation was estimated according a score based on 5 point: none, mild, mild/moderate, moderate, moderate/severe, and severe inflammation [54,55].

### 2.10. Cashew Nuts Nutritional Composition

Nutritional composition of Cashew kernel samples (*Anacardium occidentale* L.) obtained from Burkina Faso, a landlocked country in West Africa, was previously detected [30]. Briefly, 100 g of cashew kernel samples containing moisture 4.86 g, protein 21.01 g, lipids (total) 44.70 g, dietary fiber (total) 3.86 g, sugars (total) 32.80 g, ash 2.68 g, and total phenols 69.64 mg.

### 2.11. Estimation of Oxidant Levels

At the end of the experiment, through the dichlorodihydrofluoresceindiacetate (H_2_DCFDA) staining method we measured the intracellular oxidant levels as previously described [56,57]. Briefly, we dissolved H_2_DCFDA probes (Invitrogen Corporation; Carlsbad, CA, USA) in a solution of ethanol with a final concentration of 12.5 mM and we kept it at −80 °C in the dark. Before use, the solution was diluted with potassium phosphate buffer with a final concentration of 125 μM. To obtain the fluorescence reactions, 96-well black microplates were loaded with potassium phosphate buffer to a concentration of 152 μM/well. Then 8 μL diluted tissue homogenate and 40 μL (152 μM dye) were added to get a final concentration of 25 μM. The variation in fluorescence intensity was monitored every 5 min for 30 min with excitation and emission wavelengths set at 485 nm and 538 nm [58].

### 2.12. Western Blots Analysis for 5-LOX and Cox-2

Western blot examination on cytosolic fraction of the paw tissue was prepared as previously described [59]. Membranes were incubated with anti-5-LOX (1:1000) (Santa Cruz Biotechnology, Heidelberg, Germany), anti-Cox-2 (1:1000) (Santa Cruz Biotechnology, Heidelberg, Germany), and β-actin (1:500) (Santa Cruz Biotechnology, Heidelberg, Germany) for the standardization. Signals were identified with enhanced chemiluminescence (ECL) detection system reagent and the relative expression of the protein bands was measured by densitometry with BIORAD ChemiDocTM XRS+software (Bio-rad, Milan, Italy). A representation of blot signals were imported to analysis software (Image Quant TL, v2003).

### 2.13. Reagents

All other materials were purchased from Sigma-Aldrich Co. Stock solutions were prepared in nonpyrogenic saline (0.9% NaCl, Baxter Healthcare Ltd., Thetford, Norfolk, UK).

### 2.14. Data Analysis

All values are expressed as mean ± standard error of the mean of N observations. For in vivo experiments, N represents the number of animals. For experiments involving histology, the photos shown are demonstrative at least three experiments performed on different experimental days on tissue sections collected from all animals in each group. The results were analyzed by two-way ANOVA when the effect of the treatment was investigated in time-dependent mode or by one-way ANOVA when the means of two or more samples were analyzed. All analysis were followed by a Bonferroni post-hoc test for multiple comparisons. In all statistical studies GraphPad Software Prism 8 (La Jolla, CA, USA) was used. A *p* value of less than 0.05 was considered significant. # *p* < 0.05 vs CAR; ## *p* < 0.01 vs CAR; ** *p* < 0.01 vs sham; *** *p* < 0.001 vs sham.

## 3. Results

### 3.1. Effect of Cashew Nuts on CAR-Induced Inflammation and Pain

**Figure 1:**
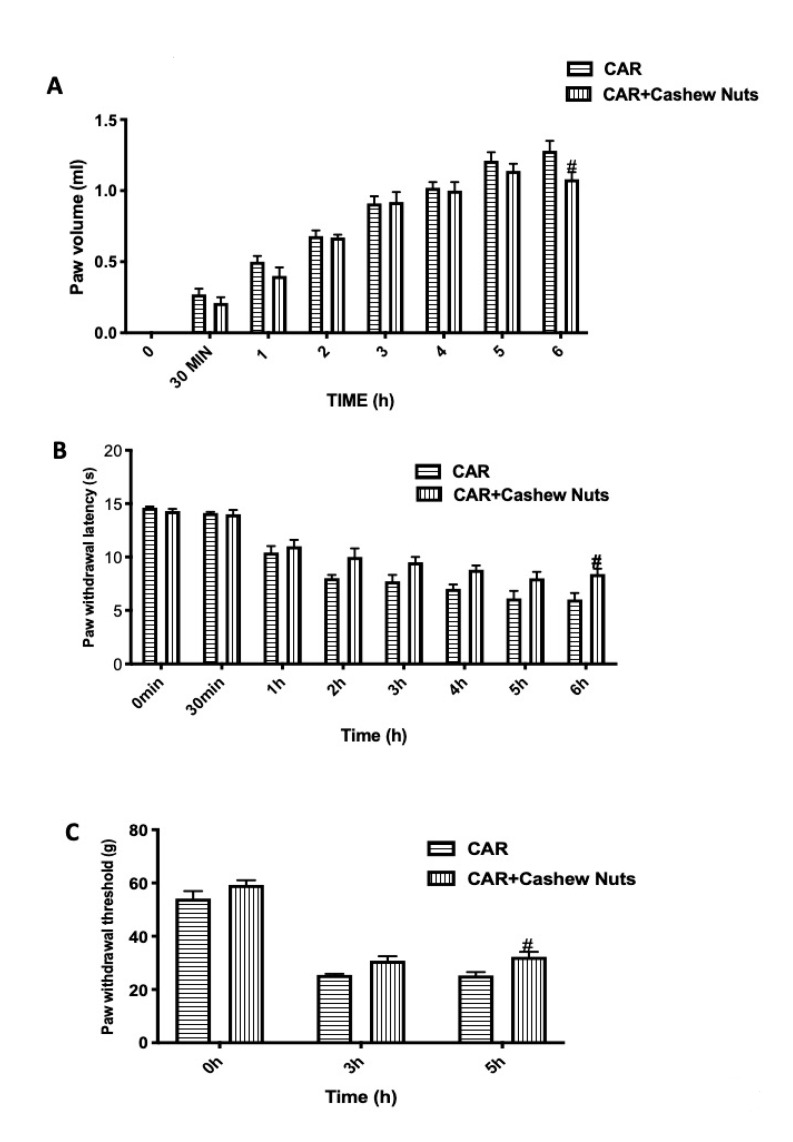
Evaluation of the effects of cashew nuts on carrageenan (CAR)-induced inflammation and pain. Paw edema was induced by subplantar injection of CAR. Paw volume was measured before the subplantar injection and hourly to 6 h. The edema volume is the difference in the paw volume at each time-point and the basal paw volume. Hyperalgesia and mechanical allodynia were assessed at the time points indicated with plantar and Von Frey tests. Cashew nuts administration shows significant improvement in the treatment of inflammation and pain. See materials and methods for further details. Paw volume (**A**); plantar test (**B**); Von Frey test (**C**). # *p* < 0.05 vs. CAR.

One of the first sign of intraplantar injection of CAR, was the increase in paw volume in a time-dependent way (Figure 1A) measured at 8 different set point from 0 (time when the experiment started) to 6 h (time when experiment ended). The increase in paw volume leads to pain that was assessed by the development of thermal hyperalgesia (Figure 1B) and mechanical allodynia (Figure 1C). In our study, we found that oral treatment with Cashew nuts at the dose of 100 mg/kg given 30 min before CAR, showed a reduction of the volume of rat paw significantly at 6 h post-CAR as well as a decrease in pain showing an inflammatory activity and algesic response.

### 3.2. Effects of Cashew Nuts on Histological Alteration after CAR Injection

**Figure 2:**
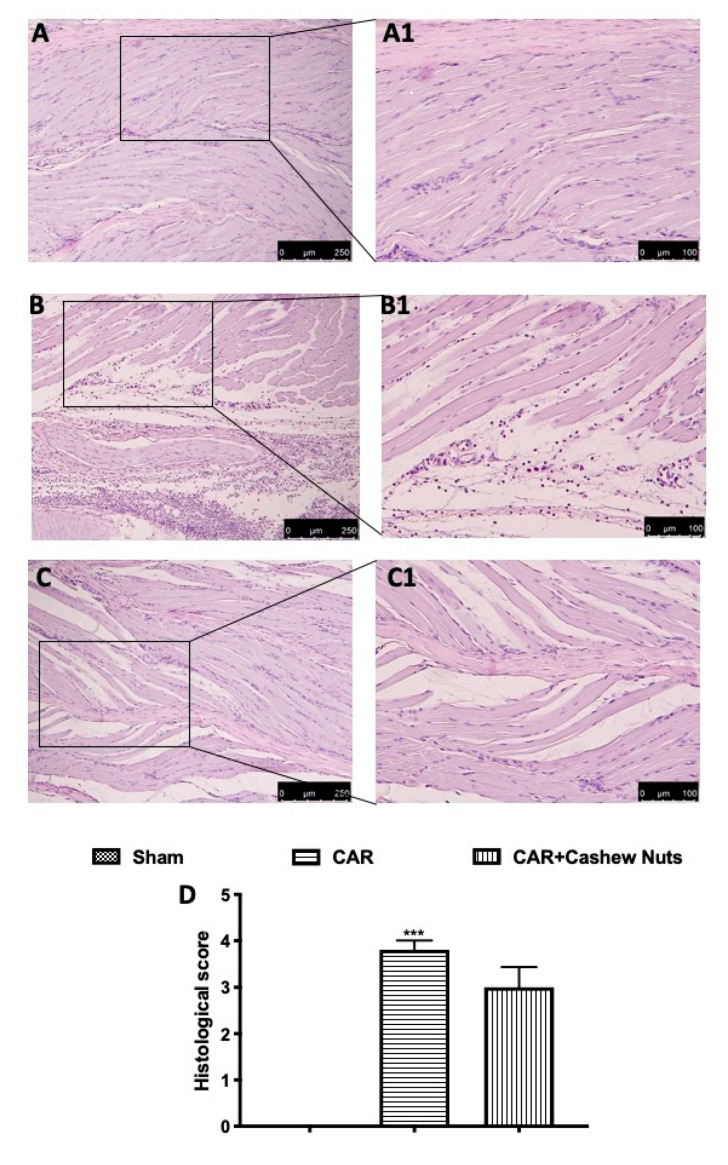
Histological evaluation of paw tissue after cashew nuts treatment following CAR-injection. Investigation of tissue injury of the animals subjected to CAR, was made by H/E staining. CAR group shows a loss of the physiological architecture compared to sham. Cashew nuts administered 30 min before CAR-injection shows reduction in infiltrating cells and edema formation compared to vehicle. Histological score (**D**). See materials and methods for further details. Sham (**A**,**A1**); CAR (**B**,**B1**); cashew nuts (**C**,**C1**). *** *p* < 0.001 vs. sham.

**Figure 3:**
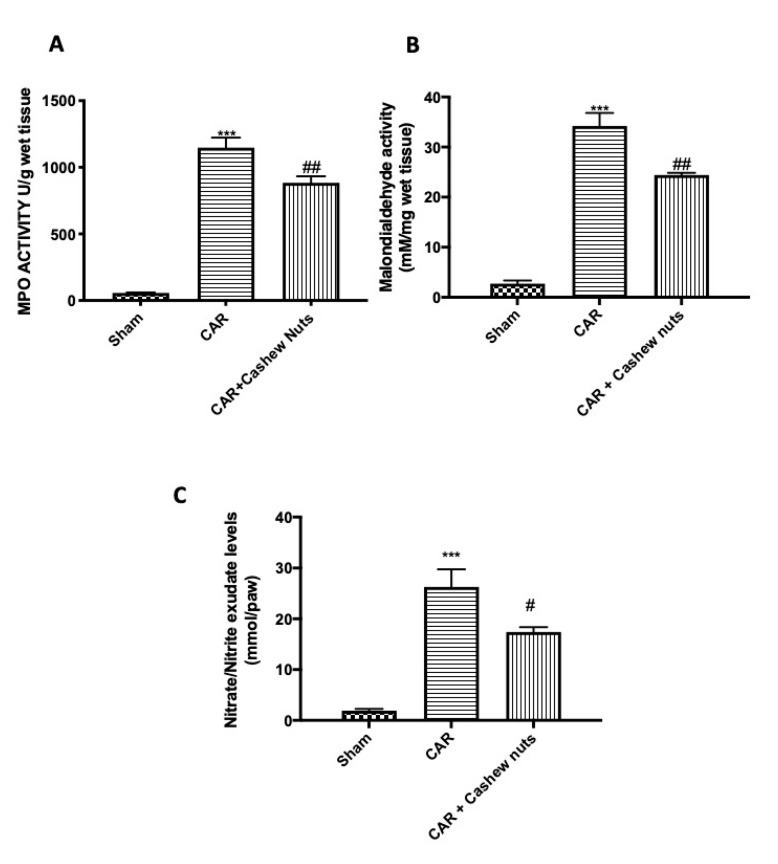
Effects of cashew nuts on nitrate/nitrite, MPO, and MDA activity after CAR-injection. As a consequence of neutrophils infiltration and lipid peroxidation MPO and MDA were assessed. Additionally, considering the key role played by NO during inflammatory events, we analyzed also levels in nitrite/nitrate in paw exudate. Carrageenan induces a significant increase in all parameter taken in consideration. On the other hands, oral treatment with cashew nuts at the dose of 100 mg/kg significantly reduced MPO, MDA, and nitrate/nitrite CAR-induction. MPO (**A**); MDA (**B**); nitrate/nitrite (**C**). See materials and methods for further details. # *p* < 0.05 vs. CAR; ## *p* < 0.01 vs. CAR; *** *p* < 0.001 vs. sham.

At the end of experiment, a histopathological study was made in paw tissue by H/E examination. A microscopic study of the paw biopsies in CAR group showed edema formation and cellular diffuses infiltration with serious alteration in tissue architecture (Figure 2B and inset B1, see histological score D). Cashew nuts administration, at the dose of 100 mg/kg, was able to slightly reduce histological injury in paw tissues of rats (Figure 3C, and inset C1, see histological score D) counteracting both cellular infiltration and edema formation. Sham rats showed a normal architecture of paw tissue (Figure 2A and inset A1).

### 3.3. Effects of Cashew Nuts on Nitrate/Nitrite, MPO, and MDA Activity in CAR-Injured Rats

The development of histological damage was associated with a statistically significant increase in MPO activity (Figure 3A), as an indicator of neutrophil infiltration, and MDA (Figure 3B), marker of lipid peroxidation. In our study, we found that cashew nuts administered 30 min before CAR injection was able to reduce not only MPO activity by inhibiting neutrophil recruitment but also MDA levels.

Additionally, during inflammatory events, NO played a critical role in tissue injury [60]. For this reason, nitrite/nitrate levels were measured in exudate of paw tissues to regulate the expression of nitric oxide (Figure 3C). Oral treatment with cashew nuts at the doses of 100 mg/kg were able to significantly decrease also nitrite/nitrate levels.

### 3.4. Effects of Anacardium occidentale L. on Cytokines Production

**Figure 4:**
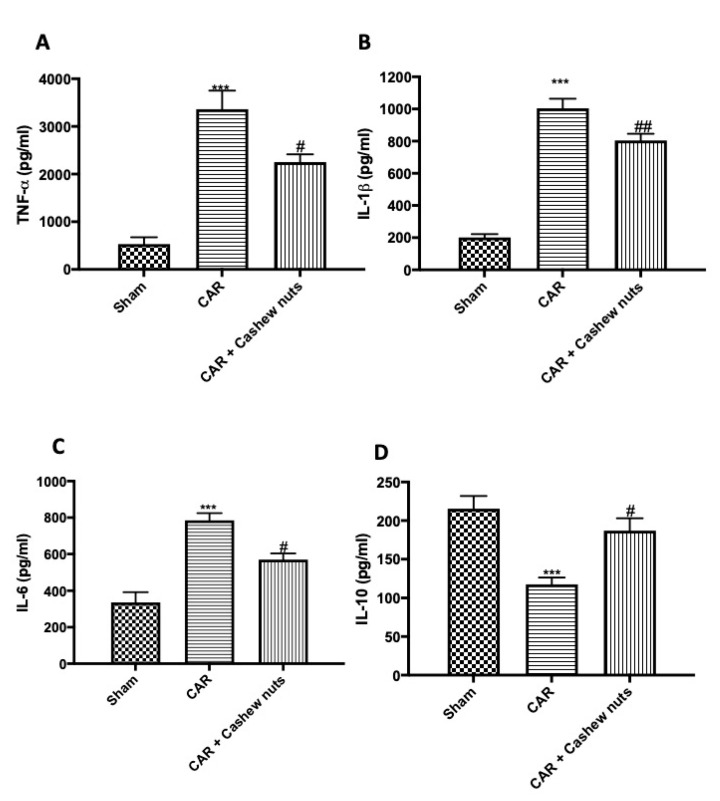
Effects of cashew nuts on cytokines production CAR-induced. Investigation of cytokines is mandatory when talking of inflammatory condition. As predicted, during CAR-caused inflammation, we observed a significant increase in TNF-α, IL-1β, IL-6 levels; vice versa we observed a significant decrease in IL-10 production. Cashew nuts administration 30 min before CAR-injection at the dose of 100 mg/kg significantly reduced pro-inflammatory cytokines expression and, vice versa, increased anti-inflammatory expression of IL-10. TNF-α (**A**), IL-1β (**B**), IL-6 (**C**), and IL-10 (**D**). See materials and methods for further details. # *p* < 0.05 vs. CAR; ## *p* < 0.01 vs. CAR; *** *p* < 0.001 vs. sham.

As well-know, cytokines exert important effects during inflammatory events, for this reason they can be used as biomarkers in indicating or monitoring inflammation and its progress [61]. In our study, we found a significant increase compared to sham animals in serum pro-inflammatory cytokine levels in the group subjected to CAR (Figure 4A–C) as well as, a significant decrease in IL-10 production was detected (Figure 4D). Cashew nuts administration given 30 min before CAR-injection at the dose of 100 mg/kg was able to significantly decrease pro-inflammatory cytokines production, and on the other hand, significantly increase IL-10 release.

### 3.5. Effect of Cashew Nuts on CAR-Induced Oxidative Stress

**Figure 5:**
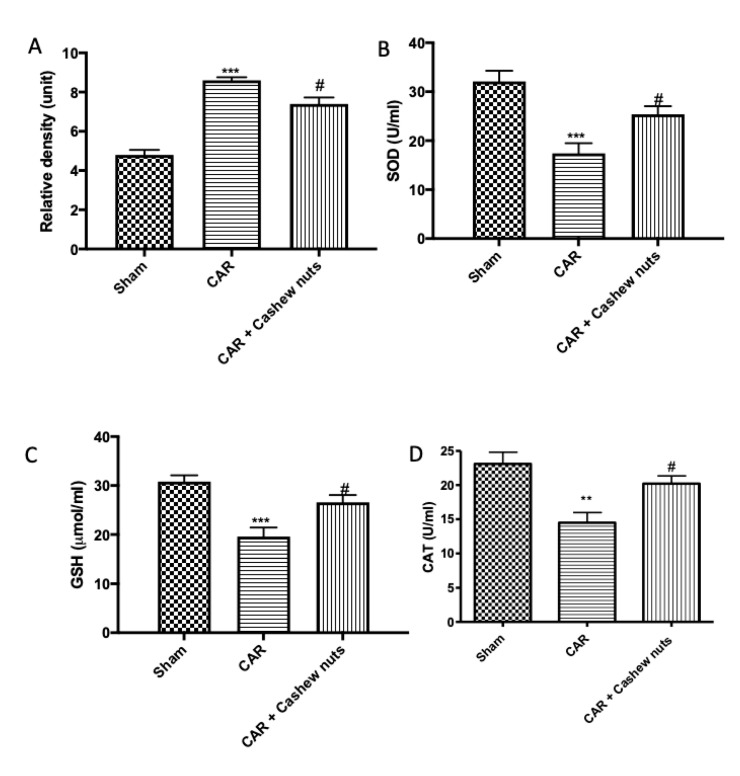
Anti-oxidant effects of cashew nuts after CAR-induction. Oxidative stress is a major component of acute inflammatory condition. First line of defense against free radical production (**A**) is represented by SOD (**B**), CAT (**C**), and GSH (**D**). Administration of cashew nuts at the dose of 100 mg/kg significantly increased the activity of the anti-oxidant enzymes SOD, CAT, and GSH which had been significantly reduced by carrageenan injection. SOD (**A**), CAT (**B**), and GSH (**C**). See materials and methods for further details. # *p* < 0.05 vs. CAR; ** *p* < 0.01 vs. sham; *** *p* < 0.001 vs. sham.

Considering a variety of oxidants and free radicals that are implicated in the pathogenesis of inflammatory process and considering that dietary components also may contribute to the antioxidant defense either by providing redox active compounds that can directly scavenge or neutralize free radicals, we investigated the oxidative stress through H_2_DCFDA probes and ELISA kits. As supposed, after CAR-injection, we observed a very important increase of ROS production (Figure 5A) and, on the other hand, a decrease in SOD (Figure 5B), GSH (Figure 5C), and CAT (Figure 5D) activity compared to sham animals. After cashew nuts treatment, decrease in oxidative stress and increase in the activity of SOD, GSH, and CAT were observed.

### 3.6. Effect of Cashew Nuts on CAR-Induced 5-LOX and Cox-2 Expressions

**Figure 6:**
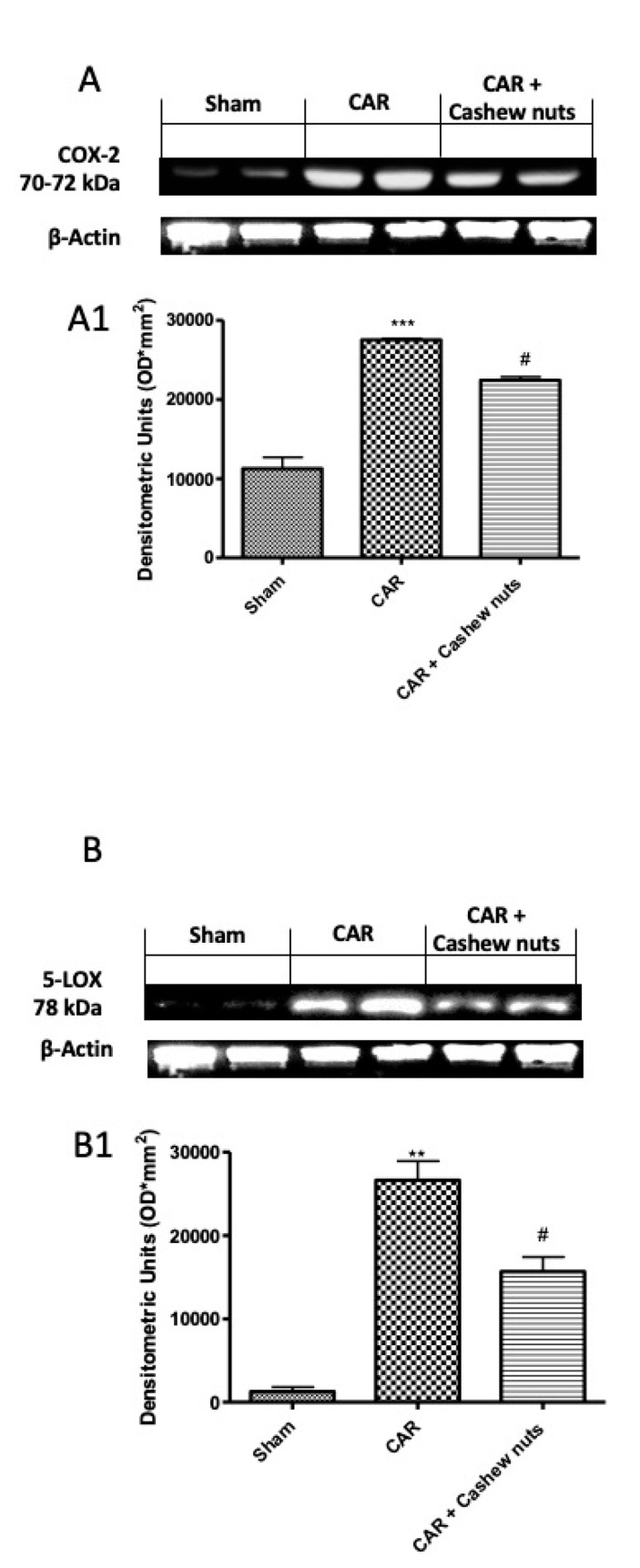
Effects of cashew nuts on 5-LOX and Cox-2 expressions. The inhibition of the biosynthesis of inflammatory mediators through the modulation of the activities of 5-LOX and/or Cox-2 considered as a promising approach to treat inflammatory diseases. For these reason we investigated the effect of cashew nuts treatment by Western blots on both enzymes. We found a significant increase after CAR injection, compared to sham, that was significantly decreased in 5-LOX as well as in Cox-2 expression. Representative western blot for Cox-2 (**A**) and LOX-5 (**B**) and respectively densitometric analysis (**A1**,**B1**). See materials and methods for further details. # *p* < 0.05 vs. CAR; ** *p* < 0.01 vs. sham.

One of the most important role, during inflammatory events, is done by the mediators of the arachidonic acid cascade from COX and LOX pathways, that, as well-known, are modulated by flavonoids [62]. For better understanding the molecular mechanism of cashew nut, we investigate by Western blots, 5-LOX and Cox-2 expressions. As speculated, after CAR injection we found a significant increase in both expression, compared to sham animals. After oral treatment with cashew nuts at the dose of 100 mg/kg, we found a significant decrease in both (Figure 6A,B).

## 4. Discussion

Inflammatory condition are universally identified as one of the most important causes of co-morbidity across the population [63]. When under control, inflammation is a defensive response of a body against invasion by the foreign bodies [64]. An acute inflammatory response is, for definition, represented by redness, heat, swelling, pain, and the loss of function [65,66]. The protective effects of inflammatory cascade and potential for tissue destruction are usually balanced in normal state, whereas, when uncontrolled, inflammation may arise in numerous diseased states like rheumatoid arthritis, multiple sclerosis, inflammatory bowel disease, and many others [4,67,68,69,70].

Considering doubts about the side effects of repeated use of synthetic chemicals, there is growing interest in the medicinal uses of natural chemicals and their derivatives as healthier replacements, such as functional products or as nutraceuticals [71].

Until today, the most advantageous therapies for the management of inflammatory state is based on the use of NSAIDs. Unfortunately, the chronic use of NSAIDs is connected with a broad spectrum of side effects ranging from gastrointestinal problems to kidney toxicity [64]. Toxicity and reappearance of signs is a major problem related to currently available synthetic drugs [64]. For these reasons the development of safer anti-inflammatory agents remains to be a subject of great interest [16]. Improvement of anti-inflammatory drugs derived from natural sources is the rational and productive strategy toward the cure of inflammatory ailments [72,73].

The search for natural molecules with antioxidant and anti-inflammatory activities has increased extremely over the past decades because the natural products are safe, efficacious, biocompatible, and cost-effective alternatives to treat inflammatory diseases [64].

In particular, researchers focused the attention on the possibilities that dietary daily intake of sources of antioxidants may offer a cost-effective approach to treating most linked pathways associated with inflammation: the oxidative stress [62,63,64].

Nuts would be a promising alternative in reducing oxidative damage, owing to their secondary metabolites richness such as polyphenols, flavonoids, tannins, terpenoids, and anthraquinones [74,75,76,77,78]. The most accredited hypothesis may be that polyphenolic components of dietary plants may increase the endogenous antioxidant defense potential and thus modulate cellular redox state, additionally, and sequentially is apt to consider how polyphenols may modulate the redox system and its components in a cell during normal and pathophysiological conditions [78].

*Anacardium occidentale* L. is a Brazilian plant that is usually consumed in nature and used in folk medicine with high value edible nut and a source of carbohydrates, proteins, phosphorous, iron, zinc, magnesium, fibers, and fatty acids [79]. Actually, it is officially listed in the National System of Medicinal Plants and Herbal Medicine of the specific Italian health system for medicinal purposes [30]. Also, it is a tree rich in anthocyanins, carotenoids, flavonoids, and other polyphenols as well as mineral components [30]. In recent years, it was used for its antioxidant, antigenotoxic, antimutagenic, antiulcerogenic, anti-inflammatory, antibacterial, antifungal, and larvicides activities [30,80,81,82,83,84,85,86,87]. In another studies made in our laboratory we demonstrated, in a murine model of colitis, that cashew nuts treatment, was able to alleviate the clinical signs of colon damage as well as oxidative stress, inflammation, and iNOS, ICAM-1, and P-selectin expressions [31].

Until today, nobody demonstrated the effect of cashew nuts treatment in an acute inflammatory model.

Carrageenan-induced paw edema is a very sensitive and reproducible test used in the screening of new molecules with anti-inflammatory activities [88]. Carrageenan-induced inflammation causes an acute and local inflammatory response that is advantageous for detecting orally active anti-inflammatory agents; therefore, it has significant prognostic value for anti-inflammatory agents acting through mediators of acute inflammation [88].

First step of acute inflammatory response is characterized by edema often formed because of exudation of fluid and plasma proteins [89]. In our work we found that edema formation was reduced significantly at 6 h post-CAR. 

Additionally, carrageenan-induced paw edema leads to sensitization of primary sensory neurons, essentially event to inflammatory pain [90]. In humans, this nociceptor sensitization usually leads to clinical conditions known as hyperalgesia defined as an increased response to a painful stimulus or allodynia described as pain evoked by non-noxious stimuli. In our study, we have proven that oral administration of cashew nuts 30 min before CAR, was in grade to reduce hyperalgesia and allodynia significantly at 6 h post-CAR.

Edema and pain in the hind paw of animals as a result of CAR-induced inflammation usually limit their motility and cause trouble in using their hind paw.

During a CAR-induced acute inflammation event, paw tissue loses normal muscle architecture and shows important amassing of infiltrating inflammatory cells and increased inter-fiber space during microscopic observation [6]. During our study, we found that oral administration of cashew nuts decreased infiltrating inflammatory cell as also demonstrated by the significant decrease of MPO assay.

One of the most dangerous consequences of uncontrolled oxidative is cell injury caused by ROS [91]. Since it is complex measuring the free radicals directly in vivo, it is common in use to carry out the quantification of different molecules which can react with these free radicals, such as for example lipids [92]. Considering that lipid peroxides are very reactive compounds, they appear to quickly degrade in a range of sub-products. MDA is one of the most known secondary products of lipid peroxidation, and it is the most used as marker of cell membrane injury [92]. Additionally, another important biomarker, in the pathogenesis of inflammation, is NO that is produced by inducible nitric oxide synthase during the formation of l-citrulline from l-arginine [93]. In our studies we found that cashew nuts at the dose of 100 mg/kg was in grade to significantly diminished lipid peroxidation and NO formation.

Conversion of arachidonic acid to biologically active leukotrienes, a potent mediators of inflammatory reactions is a key point when looking for new molecules that can inhibit inflammatory events. Plant domain is a valuable source for new 5-LOX and dual 5-LOX/COX-inhibitors, because they are fundamentally rich in flavonoids compound [36]. In past, Wagner and colleagues demonstrate that *Anacardium occidentale* L. strongly inhibited prostaglandin synthase, but nobody evaluated these activities in vivo [94]. Our results demonstrate that cashew nuts decrease inflammation probably across to the modulation of 5-LOX and Cox-2.

Inflammatory cascade activates cells and induce production of inflammatory cytokines, such as IL-1β, IL-6, and TNF-α. These molecules can potentially serve as biomarkers for diseases diagnosis, prognosis, and therapeutic management [95,96,97]. In these work we demonstrated that cashew nuts could inhibit the production of the cytokines involved in carrageenan-induced paw edema.

To counteract ROS formation, cells have developed a complex antioxidant defense system that consists of several enzyme systems involved in the conversion of ROS to less reactive molecules such as O_2_ and water [98,99]. The first line of antioxidants defense was composed by SOD, CAT, and GSH.

SOD, is the most powerful antioxidant enzyme in the cell that catalytically converts superoxide radical or singlet oxygen into hydrogen peroxide and molecular oxygen; CAT catalyzes the degradation or reduction of hydrogen peroxide to water and molecular oxygen, completing the detoxification process initiated by SOD, and GSH is the most abundant intracellular non-protein thiol in cells with the functions of removing potentially toxic electrophiles and metals protecting cells from toxic oxygen products [100,101].

In our studies, carrageenan significantly increased ROS production and reduced GSH, SOD, and CAT levels, but this increase/decrease was counteracted by the oral treatment of cashew nuts, suggesting that the inhibition of carrageenan-induced oxidative stress may also explain the analgesic effect.

## 5. Conclusions

Inflammation studies have been one of the main hubs of global science study. The inflammation is known to be correlated with oxidative processes, mainly because they share some common pathways. Since oxidative stress is common in several degenerative disease, it has been supposed that dietary antioxidants may explain a very important protective effect. Nuts are a main source of antioxidants in the diets worldwide. Nuts are high in antioxidant, in fiber, and in beneficial unsaturated fats and low in saturated fats. Nuts are usually eaten as a snack or added to food to provide both nutrients and bioactive antioxidants. In conclusion, in our work, we demonstrated for the first time that cashew nuts consumption not only brings benefits in experimental mouse models of chronic inflammation, but also in acute inflammation events. In particular was in grade to significantly counteract edema formation and consequently carrageenan-related pain. In addition, oral treatment with 100 mg/kg of cashew nuts significantly decreased MPO and MDA activity as well as nitrate/nitrite formation. Moreover, in agreement with our previous study, we demonstrated for the first time, that cashew nuts administration was able to significantly improve endogenous antioxidant activity, limiting pro-inflammatory cytokines release. Its beneficial effect is probably due to the high content of phenols that mediate activation of 5-LOX COX pathways. Considering all the benefits brought by cashew nuts, its usual consumption in the diet could be considered in order to reduce the events of cellular oxidative stress. Taken together, our result fits with previous study in which it was demonstrated that cashew nuts possess interesting anti-inflammatory, anti-oxidative, and analgesic activities that will be of interest for further investigation.
